# Perception Accuracy of Affiliative Relationships in Elementary School Children and Young Adolescents

**DOI:** 10.3389/fpsyg.2017.01936

**Published:** 2017-11-03

**Authors:** João R. Daniel, Rita R. Silva, António J. Santos, Jordana Cardoso, Leandra Coelho, Miguel Freitas, Olívia Ribeiro

**Affiliations:** ^1^William James Center for Research, ISPA – Instituto Universitário, Lisbon, Portugal; ^2^Social Cognition Center Cologne, University of Cologne, Cologne, Germany

**Keywords:** accuracy, dyadic relationships, elementary school children, young adolescents, social cognitive maps, stochastic actor-based models

## Abstract

There has been a rapid growth of studies focused on selection and socialization processes of peer groups, mostly due to the development of stochastic actor-based models to analyze longitudinal social network data. One of the core assumptions of these models is that individuals have an accurate knowledge of the dyadic relationships within their network (i.e., who is and is not connected to whom). Recent cross-sectional findings suggest that elementary school children are very inaccurate in perceiving their classmates’ dyadic relationships. These findings question the validity of stochastic actor-based models to study the developmental dynamics of children and carry implications for future research as well as for the interpretation of past findings. The goal of the present study was thus to further explore the adequacy of the accuracy assumption, analysing data from three longitudinal samples of different age groups (elementary school children and adolescents). Our results support the validity of stochastic actor-based models to study the network of adolescents and suggest that the violation of the accuracy assumption for elementary school children is not as severe as previously thought.

## Introduction

There is a long tradition in developmental research in studying the mechanisms that give rise to particular peer network structures (i.e., the enduring pattern of interpersonal relationships between children/adolescents within a specific setting, typically a school or a classroom) and how they afford and constrain behavior (for reviews see Vaughn and Santos, 2009; Santos and Vaughn, in press). Different attempts have been made to describe the essential features of these structures and recent advances in social network analysis have led to a rapid growth of studies focused on selection and socialization processes of dyadic relationships (e.g., Schaefer et al., 2010; Daniel et al., 2013, 2016; Dijkstra et al., 2013; Martin et al., 2013; Sijtsema et al., 2014). Probably the most relevant of these advances was the development of stochastic actor-based models to analyze longitudinal social network data (for an introduction to these models, see Snijders et al., 2010). These models allow estimating parameters that express the influence of different network/endogenous and covariate/exogenous processes in promoting change in dyadic relationships (i.e., in creating new relationships or terminating others). Endogenous processes determine how the structure of the network itself influences change (i.e., how existing dyadic relationships act as a catalyst of change), while exogenous processes determine how individual or dyadic characteristics influence change.

Despite the proliferation of studies using stochastic actor-based models, recent findings (Neal et al., 2016) have cast a shadow on one of the core assumptions of these models, the assumption that actors have an accurate knowledge of the dyadic relationships within the network (Snijders et al., 2010, p. 46, assumption 3) – that is, actors correctly know who is connected to whom. Neal et al. (2016) collected cognitive social structures data (CSS; Krackhardt, 1987) to test whether elementary African American school children (aged between 7 and 11 years old) have accurate perceptions of their classmates’ relationships. The authors provided each participating student with a complete class roster and asked her/him to circle the names of all of the classmates that each student (including themselves) hanged out with often. The authors found a low agreement (Cohen’s *k* = 0.371; Cohen, 1960) between what children in their sample perceived to be two classmates who hanged out together and what “truly” happened (reciprocated self-reported hang out relationships were used as the criterion to identify “true relationships”; see more details on this operationalization below). These results led the authors to conclude that elementary school children are very inaccurate in correctly identifying with whom their classmates hang out with, and thus stochastic actor-based models may not be appropriate to analyze longitudinal social network data.

In practice, the network processes one chooses to include in stochastic actor-based models determine whether actors need to be knowledgeable of all network members’ dyadic relationships or, less restrictively, whether they only need to be knowledgeable of the dyadic relationships of those to whom they are connected to (see Snijders et al., 2010 for a more detailed discussion of the basic assumptions of these models). The less strict assumption usually applies. However, Neal et al. (2016) showed that in their sample, children’s perception accuracy of the relationships involving those they affiliated with was even worse than the perception accuracy of the relationships involving those they did not affiliate with. This means that in their study even the less restrict knowledge assumption was severely violated.

Given the widespread use of stochastic actor-based models to analyze peer network dynamics, Neal et al.’s (2016) results have deep implications for future research in this area as well as for the interpretation of past findings. It is therefore important, particularly for those who rely on stochastic actor base-models to study social development, to further analyze the adequacy of the accuracy assumption that stochastic actor-based models incorporate, testing it for other age groups and different peer report methods.

### The Present Study

In this study, we wished to extend Neal et al.’s (2016) cross-sectional findings to three Portuguese longitudinal samples of different age groups and to a different peer report method. Our samples include seventh graders followed in three consecutive years (Sample 1, three waves of data collection), second graders followed in three consecutive years (Sample 2, three waves), and second to fourth graders followed in two moments in the same school year (Sample 3, two waves).

Neal et al. (2016) used CSS (Krackhardt, 1987; see also Newcomb, 1961) to assess both children’s perceptions of affiliative relationships and “true relationships” (reciprocated self-reports). Here, we attempted to extend their results to a different data collection technique. Thus, in the present study, in alternative to CSS we used social cognitive maps (SCM; Cairns et al., 1985) to assess both children’s perceptions and “true relationships.” Below we describe these two peer report methods and highlight their differences.

### Assessing Perceptions of Relationships

Cognitive social structures and social cognitive maps both assess individual perceptions of social network structures, but even though both have been used in developmental research to study peer groups (Neal, 2008; Gest and Kindermann, 2012), SCM are much more widespread among developmental researchers.

Krackhardt (1987) developed CSS as a method to assess individuals’ perceptions of the network structure (within a bounded social system) and presented his work in a *Social Networks* paper using data from a management team of a small manufacturing firm. Krackhardt (1987) asked his participants to fill out a questionnaire with a series of items about “*who goes to whom for help and advice*” (p. 118). Bellow each question (e.g., “*Who should Steve Boise go to for help or advice at work?*”; p. 118) he provided a list containing the names of the remaining managers and participants were asked to check their answers. The same question was then repeated for every manager. Krackhardt’s understanding of social network structures was that perceived networks and actual networks were conceptually distinct, and that both were phenomena of interest in their own right. Krackhardt’s (1987) concluding remarks, where he states that “*very little effort was made to ground the data presented here in behavioral terms*” (p. 128) highlight this point.

Cairns et al. (1985), Cairns and Cairns (1994), and Gest et al. (2003) believed that children were capable of providing reliable information about members of subgroups (within the larger peer group) beyond the one they belonged to. SCM were developed to deal with the limitations of the methods that were available at the time to collect *complete* social network data in school settings where student participation rates are usually far from 100% (Cairns et al., 1985; Cairns and Cairns, 1994; Gest et al., 2003). The authors’ goal was to obtain data that was a valid approximation of dyadic affiliative patterns. Cairns et al. (1985) presented their work in the *Journal of Early Adolescence* using longitudinal data of seventh to ninth graders. They tape-recorded interviews where adolescents were asked to freely recall the names of all classmates (including themselves) who hanged around together a lot, and the names of those who did not hang around with any particular group. The goal was to create “*a procedure for assessing an adolescent’s status in a particular social system, including the identity of the specific persons with whom he/she was affiliated*” (Cairns et al., 1985; p. 340). As is clear from this quote, the authors were not interested in the conceptual distinction (as was Krackhardt, 1987) between actual and perceived social structures, and they actually showed that perceived social groups (1) showed high levels of consensus between participants and were closely related to the occurrence of (2) positive behavioral interchanges and of (3) sociometric friendship nominations (see also Gest et al., 2003).

In more practical terms, when collecting data in school settings, the main difference between CSS and SCM is that, while CSS require participants to identify for each classmate at a time with whom s/he hangs out with, SCM require participants to identify groups of classmates who hang around together (or who do not hang around with anyone). As far as we know, no study has yet compared the structure of individual and aggregated CSS and SCM to see how much they match each other. Nevertheless, data collected from both methods can be analyzed in equivalent manners. Furthermore, social structures, where the different subgroups (identified in SCM) are embedded, emerge from the development of dyadic relationships. Also, while producing his/her SCM, students can identify subgroups of any size, including size of two (i.e., dyads).

Because perceptions of peer relationships are theoretically important to explain how network structures arise, the relevance of this study goes beyond the methodological implications on how we collect and analyze peer reports of social relationships, and their relation to the accuracy assumptions of stochastic actor-based models (and consequent validity of such models). Agency-based accounts of network structure claim that perceptions about relationships, and not the actual network structure itself, drive individual behavior toward others (Krackhardt, 1987; Brands, 2013). Also, there is evidence that suggests that children with more accurate perceptions of social relationships are better adjusted (Crick and Dodge, 1994; Cillessen and Bellmore, 1999; Bellmore and Cillessen, 2003; Morrow et al., 2016). Children’s ability to successfully navigate the social world of peers thus seems to depend on how well they perceive social structures (Cappella et al., 2012).

## Materials and Methods

### Participants

This study includes data from three longitudinal samples from urban schools in the Lisbon metropolitan area, Portugal. In the Portuguese school system, children and adolescents are organized in self-contained classes of students that share the same schedule, space and teacher(s) throughout the year.

Sample 1 was collected between 2009 and 2013 (spring semester), Sample 2 was collected between 2012 and 2016 (spring semester), and Sample 3 was collected in 1990 (February and June). Participants provided assent and were given written consent from their parents to participate in this study. This study was approved by Comissão Nacional de Proteção de Dados (the Portuguese National Commission for Data Protection). Data on socioeconomic status was not collected from the participating students or schools, but participants were roughly characterized by the researchers who collected the data as belonging to “middle to lower middle class” in Samples 1 and 3, and “middle to higher middle class” in Sample 2. Sample 1 included a small proportion of minority children (no detailed records are available), and Samples 2 and 3 were ethnically homogenous (Caucasian).

#### Sample 1

One hundred and forty-five seventh graders (72 girls and 73 boys) from two public schools with a mean (self-reported) age of 12.49 years old (*SD* = 1.00) at the first data point. This sample is a subset of a larger sample (623 seventh graders) restricted to participants with SCM data in more than one school year in classes with response rates > 0.60 (**Table 1**). Contrary to the other samples, in Sample 1 the number of different classes to which participants belonged to varied across years due to academic retentions and some reshuffling of class rosters in consecutive years (number of different classes: year 1 = 12, year 2 = 18, and year 3 = 15; **Table 1**).

**Table 1:** Sample descriptives.

	Time 0			Time 1			Time 2		
	*N*	*M*	*SD*	*N*	*M*	*SD*	*N*	*M*	*SD*
**Sample 1 (*n* = 145)**									
Classes	12			18			15		
Participants (7G/8G/9G)	145/0/0			16/122/0			1/14/59		
Class size		24.26	2.23		22.08	3.33		21.43	3.44
Class participation rates		0.76	0.08		0.85	0.10		0.86	0.11
**Sample 2 (*n* = 40)**									
Classes	3			3			3		
Participants (2G/3G/4G)	40/0/0			0/38/0			0/0/39		
Class size		20	–		19.26	1.27		18.56	1.25
Class participation rates		0.74	0.06		0.80	0.11		0.87	0.10
**Sample 3 (*n* = 72)**									
Class	5			5					
Participants (2G/3G/4G)	10/24/38			10/24/38					
Class size		22.25	0.80		22.25	0.80			
Class participation rates		0.83	0.17		0.83	0.17			

*G, grade. All classes of Sample 2 at Time 0 were of the same size.*

#### Sample 2

Forty second graders (19 girls and 21 boys) from one private school with a mean (self-reported) age of 7.19 years old (*SD* = 0.47) at the first data point. This sample is a subset of a larger sample (97 second graders) also restricted to participants with SCM data in more than one school year in classes with response rates > 0.60.

#### Sample 3

Seventy-two elementary school children (48 girls and 24 boys) from one public school. This sample is a subset of a larger sample (201 second to fourth graders) restricted to participants with SCM data at both time points in classes with response rates > 0.60. No age data is available for this sample, although generally most second, third, and fourth graders in the Portuguese school system are 7, 8, and 9 years old (respectively) in February (first wave of data).

### Procedure

Participants were provided with a questionnaire and asked to list groups of classmates (including themselves) who hanged around together a lot and also to identify those who did not hang around with any particular group. Participants were given a list containing the names of their classmates, but questions were open-ended and participants could nominate as much or as few peers as they wished. Assessments were made in one in-classroom session. We excluded participants who failed to self-report their status regarding who they hanged around with (participation rates also reflect these excluded cases).

### Measures: Perception Accuracy

First, we transformed participants’ answers in individual symmetric nomination matrices (nomination*_ij_* = 1 – peers grouped together, nomination*_ij_* = 0 – peers not grouped together). Next, similarly to Neal et al. (2016) we used reciprocated self-reported hang around relationships as the criterion to identify “true relationships.” We then measured Overall participant’s accuracy in perceiving whether a pair of classmates were in fact related using Cohen’s *k* (Cohen, 1960). Briefly, for each participant we compared her/his nomination matrix with the class “true relationships” matrix (i.e., reciprocated self-reported hang around relationships) to calculate the number of: (a) accurately perceived affiliative relationships (A1; nomination*_ij_* = 1, relationship*_ij_* = 1), (b) accurately perceived non-relationship (A0; nomination*_ij_* = 0, relationship*_ij_* = 0), (c) false positives (FP; nomination*_ij_* = 1, relationship*_ij_* = 0), and (d) false negatives (FN; nomination*_ij_* = 0, relationship*_ij_* = 1). These proportions were then used to compute *k*:

*k* = PA – PE/(1 – PE), where

PA (proportion of agreements) = (A1 + A0)/total

PE (expected or chance agreement) = [(A1 + FP)/total] × [(A1 + FN)/total] + [(A0 + FN)/total] × [(A0 + FP)/total].

For example, if participant A reports a group including B and C, s/he is reporting that she perceives B and C to be related. Participant A report is then compared with what B and C report about themselves. If B and C both report they hang around together (i.e., if they place themselves together in a group), then A has an accurate perception of B and C relationship. If B and C do not report they hang around together, than A as an inaccurate perception of B and C relationship.

Using reciprocated self-reported hang around relationships as the criterion to identify “true relationships” makes our results comparable to Neal et al. (2016). This criterion considers the two individuals directly involved in the relationship the best informants of its status (but see for example Cairns et al., 1985; Cappella et al., 2012 for different criteria based on aggregated responses of all participants).

We computed two additional perception accuracy measures for each participant – perception accuracy of affiliates and perception accuracy of non-affiliates. One refers exclusively to the accuracy of relationships involving peers with whom participants affiliated with (reciprocated hang around self-reports), the other refers exclusively to the accuracy of relationships involving peers with whom participants did not affiliate with, respectively. Perception accuracy of affiliates’ relationships reflects how accurate participants are in identifying dyadic relationships of those they affiliate with (i.e., dyads where at least one member is an affiliate of the participant); and perception accuracy of non-affiliates’ relationships reflects how accurate respondents are in identifying dyadic relationships of peers they do not affiliate with (i.e., dyads where none of the members is an affiliate of the participant). These measures were computed similarly as above, but for affiliates’ perception accuracy we selected only individual nominations and “true relationships” matrices’ rows referring to each participant’s reciprocated hang around self-reports. For the perception accuracy of non-affiliates’ relationships, we selected only matrices’ rows and columns referring to participant’s non-affiliates (i.e., relationships involving the participant and participant’s affiliates are excluded from this measure).

### Data Analysis

All models described below refer to longitudinal multilevel regression model with repeated measures nested within participants (i.e., participants – level 2, repeated measures – level 1) and were fitted using lmerTest package in R version 3.2.3 (R Core Team, 2015; Kuznetsova et al., 2016). For Sample 1 only, we ran 3-level models with classes (level 3) as a crossed random factor (because some participants changed classes from 1 year to the next due to grade retention or to merging of different classes). For Samples 2 and 3, there were few different classes to add a third level.

First, we made cross-sample comparisons of perception accuracy measures. Next, for each sample, we ran two different models. First we ran a longitudinal multilevel regression model (random intercept, plus time fixed effect) to test for change in perception accuracy of peers’ relationships across time (Model 1). These models assume that all participants (within samples) have similar rates of change. A more realistic assumption would be to allow participants to have different rates of change (random intercept and time random slope model). But when estimating these time random slope models for Samples 1 and 2, the estimated time slope variance was 0 (Sample 3 only has two data points and as such we can only include one random effect). Consequently we opted to include only a time fixed effect in the models.

Next, we reshaped the data into long format and ran models with perception type (affiliates vs. non-affiliates) as a predictor to test for differences in the rate of change of perception accuracy of relationships involving participants’ affiliates and non-affiliates (Model 2).

To assist models’ interpretation we provide a brief description of what estimated regression coefficients represent/test in Supplementary Table 1.

## Results

Across samples the mean number of groups reported by each participant ranged from 4.67 to 6.51, and included on average from 75 to 98% of the participants’ peers (**Table 2**). Although participants could place themselves or their peers on more than one group they rarely did so (only 17 cases in total). Dyads and triads were the most common groups identified (dyads cross-sample range: 26–54%; triads cross-sample range: 19–29%; isolates are excluded for these calculations), and mean in-group size (i.e., the group where participants’ reported they belonged to; cross-sample range: 3.60–6.18) was higher than the mean out-group size (i.e., the groups where participants’ did not include themselves; cross-sample range: 2.75–3.83). Estimated mean differences were: β_Sample1_ = 1.46, *SE* = 0.12, *p* < 0.001, β_Sample2_ = 1.94, *SE* = 0.21, *p* < 0.001, and β_Sample3_ = 0.81, *SE* = 0.10, *p* < 0.001 (detailed test statistics provided in Supplementary Table 2). Dyads were over-represented in out-groups (Sample 1 = 36%, Sample 2 = 31%, and Sample 3 = 55%) compared to in-groups (Sample 1 = 11%, Sample 2 = 7%, and Sample 3 = 25%): χ^2^_Sample1_ = 169.01, *df* = 13, *p* < 0.001, χ^2^_Sample2_ = 84.13, *df* = 13, *p* < 0.001, χ^2^_Sample3_ = 83.11, *df* = 8, *p* < 0.001 (see more details in Supplementary Table 3). The difference between in-group size and the mean number of reciprocated self-reported relationships indicates that participants tended to over-estimate their in-group size. Nevertheless, the numbers of reciprocal relationships identified are comparable to those obtained with different sociometric data, albeit fort different type of relational data (e.g., friendship ties: Dijkstra et al., 2013; Sijtsema et al., 2014; like ties: Huitsing et al., 2012).

**Table 2:** Summary of individual social cognitive mappings.

	% Classmates identified		*N* groups reported		Size groups reported				In-group size		Out-group size		Reciprocated self-reported relationships	
	*M*	*SD*	*M*	*SD*	Isolates	Dyads	Triads	≥4	*M*	*SD*	*M*	*SD*	*M*	*SD*
**Sample 1**														
Time 0	0.84	0.14	6.30	1.92	232	223	134	324	5.32	2.70	3.69	2.01	2.99	1.91
Time 1	0.92	0.10	6.35	1.79	191	208	151	326	4.89	2.22	3.69	1.86	2.67	1.90
Time 2	0.94	0.06	5.89	1.60	101	87	86	161	5.97	2.94	3.75	2.02	3.35	2.20
**Sample 2**														
Time 0	0.96	0.07	4.75	1.56	14	47	39	90	6.18	2.96	3.77	1.91	2.00	1.65
Time 1	0.98	0.04	4.84	1.48	14	43	42	85	5.72	2.76	3.71	1.73	2.97	1.89
Time 2	0.98	0.04	4.67	1.22	17	41	32	92	5.45	2.59	3.83	1.89	2.15	1.57
**Sample 3**														
Time 0	0.77	0.18	6.22	2.23	2	240	94	112	3.60	1.61	2.75	1.23	1.32	1.21
Time 1	0.75	0.21	6.51	3.10	41	184	122	122	3.73	1.41	2.86	1.17	1.58	1.44

*% Classmates identified – proportion of all classmates nominated by each participant; Groups reported – number of groups reported by each participant; Group size – frequency of group types reported by all participants; In-group/Out-group – groups reported by the participants’ that include/exclude themselves (mean values exclude isolates, i.e., size = 1).*

**FIGURE 1:**
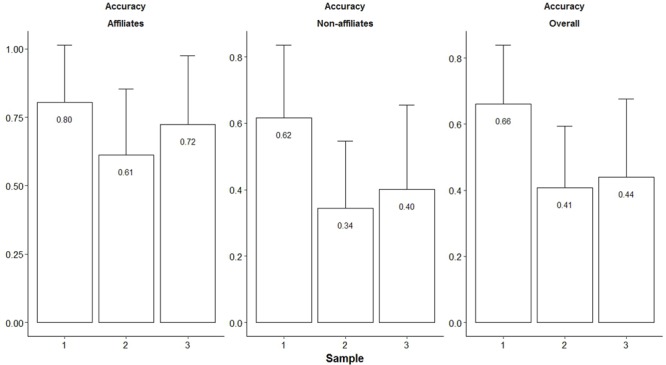
Comparisons of perception accuracy of affiliative relationships (Cohen’s *k*; *M* + *SD*) for Samples 1–3. Affiliates accuracy measure refers to the perception accuracy of the relationships involving peers that children/adolescents affiliate with; Non-affiliates accuracy measure refers to the perception accuracy of the relationships involving peers that children/adolescents do not affiliate with; Overall accuracy measure refers to the perception accuracy of all peers’ affiliative relationships.

**Table 3** presents means and standard deviations for perception accuracy measures. We found cross-sample significant differences for perception accuracy (**Figure 1**). Briefly, Sample 1 participants had higher perception accuracy than their counterparts in Samples 2 and 3 (elementary school children). Sample 2 and Sample 3 participants had similar perception accuracy, except for the relationships involving peers they affiliated with (Sample 3 participants ranked higher than Sample 2 counterparts; detailed test statistics for these comparisons are provided in Supplementary Table 4).

**Table 3:** Perception accuracy descriptive statistics.

	Time 0		Time 1		Time 2	
	*M*	*SD*	*M*	*SD*	*M*	*SD*
**Sample 1**						
Overall	0.66	0.19	0.64	0.18	0.69	0.15
Affiliates	0.82	0.21	0.79	0.21	0.80	0.19
Non-affiliates	0.61	0.24	0.61	0.21	0.64	0.19
**Sample 2**						
Overall	0.30	0.18	0.51	0.17	0.41	0.14
Affiliates	0.51	0.30	0.71	0.17	0.61	0.20
Non-affiliates	0.23	0.18	0.43	0.21	0.37	0.16
**Sample 3**						
Overall	0.40	0.27	0.47	0.22		
Affiliates	0.64	0.29	0.80	0.19		
Non-affiliates	0.37	0.16	0.40	0.24		

**FIGURE 2:**
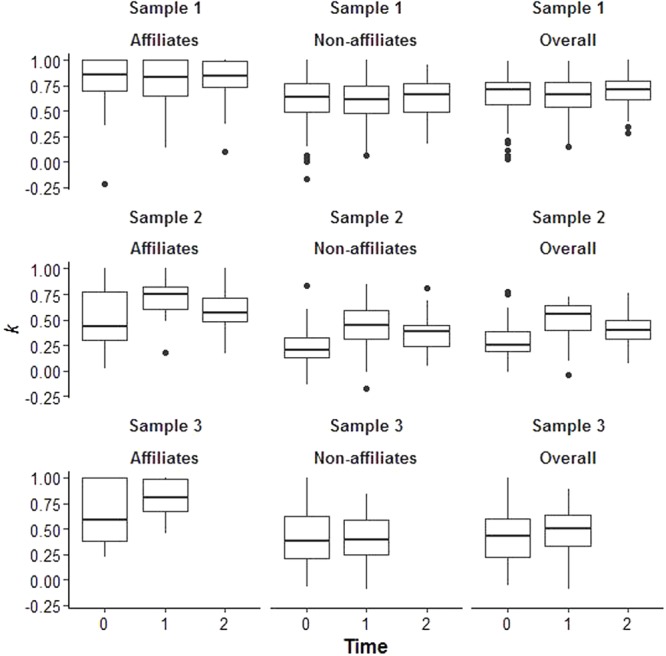
Distribution of perception accuracy (Cohen’s *k*) across time and sample. Affiliates accuracy measure refers to the perception accuracy of the relationships involving peers that children/adolescents affiliate with; Non-affiliates accuracy measure refers to the perception accuracy of the relationships involving peers that children/adolescents do not affiliate with; Overall accuracy measure refers to the perception accuracy of all peers’ affiliative relationships.

**Figure 2** illustrates the distribution of perception accuracy values. Despite some degree of variability, almost all participants had accuracy values above chance levels (*k* > 0). In the two samples of elementary school children (Samples 2 and 3) we found perception accuracy values of all peers’ relationships (“Overall” perception accuracy) to range between 0.30 and 0.51 (Sample 2 mean *k* T*_0_*–T*_2_*: 0.30, 0.51, and 0.41; Sample 3 mean *k* T*_0_*–T*_1_*: 0.40 and 0.47; **Table 3**). Neal et al.’s (2016) findings (*k* = 0.37) fall in this range. Using Cohen’s *k* reference values (0.01–0.20 none to slight, 0.21–0.40 fair, 0.41–0.60 moderate, 0.61–0.80 substantial, and 0.81–1.00 almost perfect), around 75% of Sample 2 and 3 participants showed a fair to substantial perception accuracy of peers’ relationships (**Figure 2**). In the older age sample (Sample 1) we found mean perception accuracy values to be much higher than those reported for elementary school children (Sample 1 mean *k* T*_0_*–T*_2_* range = 0.64–0.69). Again, using Cohen’s *k* reference values, around 75% of the young adolescents in our sample showed a moderate to almost perfect perception accuracy of peers’ relationships (**Figure 2**).

In all samples, accuracy of affiliates’ relationships was higher than the accuracy of non-affiliates’ relationships (**Table 4**). Perception accuracy values were stable across time for Sample 1, but not for Samples 2 and 3 (**Table 5**, Models 1 and 2 β_1_). In Samples 2 and 3 perception accuracy increased over time (**Table 5**, Models 1 and 2 β_1_), although in Sample 3 the rate of change was higher for perception accuracy of affiliates’ relationships (**Table 5**, Model 2 β_3_).

**Table 4:** Perception accuracy: affiliates vs. non-affiliates.

	Affiliates		Non-affiliates		Fixed Effect				Random Effects		
	*M*	*SD*	*M*	*SD*	β	*SE*	*p*		σ^2^ intercept	σ^2^residual	σ^2^class
Sample 1	0.81	0.21	0.62	0.22	0.19	0.02	<0.001	^∗∗∗^	0.000	0.042	0.003
Sample 2	0.61	0.24	0.34	0.20	0.27	0.03	<0.001	^∗∗∗^	0.005	0.044	
Sample 3	0.72	0.25	0.40	0.26	0.31	0.04	<0.001	^∗∗∗^	0.011	0.054	

*βs represent estimated mean differences between participants’ perception accuracy of the relationships of those they hang around with and those they do not hang around with.*

**Table 5:** Estimates of perception accuracy change across time.

	Sample 1				Sample 2				Sample 3			
	Coefficient	*SE*	*p*		Coefficient	*SE*	*p*		Coefficient	*SE*	*p*	
**Model 1: Overall**												
**Fixed effects**												
β_0_ Intercept	0.66	0.02	<0.001	^∗∗∗^	0.36	0.03	<0.001	^∗∗∗^	0.41	0.03	<0.001	^∗∗∗^
β_1_ Time	0.01	0.01	0.444		0.05	0.02	0.011	^∗^	0.06	0.03	0.070	
**Random effects**												
σ^2^ Intercept	0.002				0.001				0.024			
σ^2^ Residual	0.024				0.032				0.032			
σ^2^ Class	0.005											
**Model 2: Affiliates vs. Non-affiliates**												
**Fixed effects**												
β_0_ Intercept	0.72	0.02	<0.001	^∗∗∗^	0.42	0.02	<0.001	^∗∗∗^	0.50	0.03	<0.001	^∗∗∗^
β_1_ Time	0.00	0.01	938		0.06	0.02	0.001	^∗∗∗^	0.09	0.04	0.016	^∗^
β_2_ Affiliates	0.20	0.02	<0.001	^∗∗∗^	0.29	0.04	<0.001	^∗∗∗^	0.20	0.05	<0.001	^∗∗∗^
β_3_ Time × Affiliates	-0.01	0.02	0.480		-0.02	0.03	0.518		0.17	0.07	0.019	^∗^
**Random effects**												
σ^2^ Intercept	0.000				0.005				0.014			
σ^2^ Residual	0.042				0.042				0.050			
σ^2^ Class	0.003											

## Discussion

Recent findings reporting low perception accuracy of peer relationships have put into question the validity of stochastic actor-based models to study the developmental dynamics of social relationships in young children (aged between 7 and 11) (Neal et al., 2016). One of the core assumptions of these models is that actors have an accurate knowledge of the relationships of all members of the network (Snijders et al., 2010). In practice, a less strict assumption usually applies – actors have to be knowledgeable of the relationships of those they are connected to. But Neal et al.’s (2016) findings show that perception accuracy was worse for the relationships involving peers they affiliated with than for relationships involving peers they did not affiliate with. With the present study we aimed at testing further the adequacy of the accuracy assumption that stochastic actor-based models incorporate, extending Neal et al.’s (2016) cross-sectional analyses to longitudinal data of different age groups and with a different peer report measure.

Using longitudinal data from our two samples of elementary school children with approximately the same age range as the children in Neal et al.’s (2016) sample, we show that a more strict (stochastic actor-based models’) assumption, requiring actors to have an accurate knowledge of all the relationships in the network, might be violated for some elementary school children (particularly for younger ones), as Neal et al. (2016) suggested, but not so for young adolescents.

However, different from Neal et al. (2016), participants in all our samples were more accurate of the relationships involving peers they affiliated with than of those involving peers they did not affiliate with (*k* differences of 0.20 to 0.30, depending on sample; **Table 4**). The median values of perception accuracy of affiliates’ relationships in our samples (**Figure 2**) indicate that the more common and more relaxed assumption of stochastic actor-based models, requiring actors to have an accurate knowledge of only the relationships of the actors they share a tie with, is not severely violated for the majority of elementary school children, although there is some variation among participants’ scores. As pointed out by one reviewer, all statistical modeling assumptions are approximations, and the validity of conclusions drawn from statistical models depends on how one expects deviations from these assumptions to influence conclusions, and also on the availability of alternative methods. Given the estimates of perception accuracy of affiliates’ relationships presented above and the lack of better alternative methods to analyze the type of longitudinal network data usually collected by developmental researchers, we believe that stochastic actor-based models remain a valuable tool for studying the developmental dynamics of elementary school children’s relationships. As to young adolescents, estimated perception accuracy values raised no major concerns as to the validity of using stochastic actor-based models in this age group.

The different accuracy levels found in our and Neal et al.’s (2016) study might be related with the different peer report method that was used. Although CSS and SCM share some commonalities, these methods differ in how they access information of social relationships that is stored in memory. While CSS require participants to identify for each classmate at a time the peers s/he hangs out with, SCM require participants to identify groups of peers who hang around together. Although both methods assess representations of the peer group structure, this small difference on what participants are asked to do might be relevant. Recent studies have shown that social network information is encoded and stored in memory mostly at the triadic and group level, not at the dyadic level (Brashears, 2013; Brashears and Quintane, 2015). Therefore, asking individuals to recall social network ties dyad by dyad (when using CSS) might be more demanding for memory, potentially decreasing accuracy. Differently, when using SCM, participants can recall information more freely (i.e., they can report dyads, triads or larger groups of peers; **Table 2**), which is more akin to how social network ties seem to be encoded in the first place. This hypothesis finds support in research on human memory showing that the more the mental processes involved in the retrieval of information match the processes involved in the encoding of that same information, the better the memory performance (transfer appropriate processing; see Roediger et al., 1989; Schendan and Kutas, 2007). It would be interesting in future studies to use both CSS and SCM in different age samples and compare perception accuracy- both overall, and for affiliative and non-affiliative ties- for the two types of peer report methods. This would allow testing whether perception accuracy is indeed higher for SCM data because encoding and recall processes are better matched in this procedure.

Brashears and Quintane (2015) suggested that humans are more prone to use triadic encoding for in-group information and group-level encoding for out-group information. According to the authors, it is this group-level encoding mechanism that leads to more inaccurate perceptions of non-affiliates relationships. They suggest that out-groups may be perceived as “*composed of unified others*,” while the nuances of in-group relationships information may be encoded in a more detailed fashion, and thus produce more accurate perceptions. However, in our data, when reporting out-groups (i.e., the groups where participants’ did not include themselves) children and adolescents tended to include less children (and report more dyads, i.e., groups of size 2) than in their in-group (i.e., the group where participants’ reported they belonged to). Comparable findings have been found for friendship ties collected with CSS (Kumbasar et al., 1994; Batchelder et al., 1997). Our findings, and that of Kumbasar et al. (1994), suggest that individuals perceive their in-group to be composed of dense network of relationships, whereas the relationships within out-groups tend to be perceived as sparse (Brands, 2013). These apparent different findings in the literature should be addressed in future research.

Following the same logic, our results suggest that outgroup data is spontaneously recalled more in dyads than in triads or larger groups of peers (**Table 2** and Supplementary Table 3). Thus, given that CSS require individuals to recall social networks in dyads, this method may favor a better perception accuracy for outgroup data than for in group data, because it matches what seems to be the spontaneous recall structure of non-affiliative ties (at least according to our results). If so, this could explain why in our study children were more accurate for affiliative than for non-affiliative ties while Neal et al. (2016) found the opposite pattern. Of course that all these hypotheses warrant empirical support and can thus be a fruitful research topic, both at the methodological and theoretical level.

Different accuracy levels found across studies might also be related with the different characteristics of the samples whose data is analyzed. For instance, Neal et al.’s (2016) sample was composed of mostly low-income African American students, while our samples were composed mostly of middle-class Caucasian students and this may account for some differences that we found in theirs and ours results. There is ample research documenting the association of negative stereotypic traits to African Americans (such as “uneducated,” “criminal,” or “poor”; see Dovidio et al., 1986; Devine, 1989; Devine and Elliot, 1995). The perpetuation of these negative stereotypes has resulted in high levels of prejudice, discrimination, segregation, and social exclusion of this racial group in society (Quillian and Pager, 2001). Recent experimental studies show that exclusion experiences distort social network perception, with individuals who experience exclusion being less accurate than individuals who do not (O’Connor and Gladstone, 2015). Thus, it is possible that the experience of social exclusion underlies the differences in the perception accuracy of affiliative and non-affiliative ties found between our results and those of Neal et al. (2016). Future studies should address this hypothesis in a systematic way.

Perception accuracy of peer relationships increased over time in elementary school children of Samples 2 and 3, but not in the young adolescents of Sample 1. This might suggest that perception accuracy of relationships stabilizes faster in younger adolescents than in elementary school children, because older children are generally better, and therefore faster in perceiving who affiliates with whom. Because our data was collected after children and young adolescents had spent several months in the company of their peers, it would be interesting in future studies to collect multiple data points within the same school year, for different age groups, and test for non-linear rates of change in perception accuracy. These models would allow the comparison of growth rates and to see, for each age group, if and when perception accuracy of peer relationships stabilizes.

The fact that we used three different samples, one from a private school and two from public schools (one of them collected many years ago) might raise some comparability issues. Possible differences in educational policies might have shaped classroom features in ways that influenced how well social relationships were perceived. But the fact that we found similar results across samples suggests that no major comparability issues exist. Understanding how educational policies shape classroom features that influence how well social relationships are perceived is an interesting avenue for further research.

## Conclusion

This study analyzed longitudinal data of perception accuracy of peer relationships in elementary school children and adolescents. Our results show that, contrary to previous findings, perception accuracy of affiliates’ relationships is higher than that for non-affiliates’ relationships. These results support the validity of stochastic actor-based models to study the developmental dynamics of young adolescent relationships, and suggest that the violation of the accuracy assumption of these models for elementary school children may be not as severe as previously found.

## Author Contributions

JD and AS: Design of the studies. AS, JC, LC, MF, and OR: Data collection. JD: Data analysis. JD and RS: Interpretation of the data and manuscript drafting. AS, JC, LC, MF, and OR: Critical revision of the manuscript. JD, RS, AS, JC, LC, MF, and OR: Final approval of the version to be published.

## Conflict of Interest Statement

The authors declare that the research was conducted in the absence of any commercial or financial relationships that could be construed as a potential conflict of interest.
