# Supplementary material for: Perception Accuracy of Affiliative Relationships in Elementary School Children and Young Adolescents
Source: Front Psychol. 2017 Nov 3;8:1936. doi: 10.3389/fpsyg.2017.01936 (PMC5676090; doi:10.3389/fpsyg.2017.01936)
Supplement: Supplementary file 1 [file Table_1.PDF]

Supplementary Material

Perception Accuracy of affiliative relationships in elementary school children and young adolescents.

Supplementary Table 1. Accuracy model predictors

	**Estimated regression coefficients' meaning**
Intercept	perception accuracy at $Time_0$ (first data point)
Time	growth in perception accuracy between each time point (i.e., from $Time_{i-1}$ to $Time_i$)
Affiliates	difference between perception accuracy of affiliates' and non-affiliates' relationships at $Time_0$<br>$\beta > 0$ – perception accuracy of relationships is higher for affiliates' relationships at $Time_0$<br>$\beta = 0$ – no difference between perception accuracy of affiliate and non-affiliates' relationships at $Time_0$
Time × Affiliates	$\beta > 0$ – perception accuracy of affiliates' relationships increases more over time than perception accuracy of non-affiliates' relationships<br>$\beta = 0$ – difference between both types of perception accuracy remains stable over time

Supplementary Table 2. Comparisons of ingroup and outgroup size

	Fixed Effect				Random Effects		
	β	*SE*	*p*		σ^2 intercept	σ^2 residual	σ^2 classroom
**Sample 1**	1.46	.12	< .001	***	.000	3.990	.538
**Sample 2**	1.94	.21	< .001	***	.296	4.077	
**Sample 3**	.81	.10	< .001	***	.180	1.525	

Note. βs represent estimated mean differences between participants' ingroup and outgroup size; ingroup/outgroup – groups reported by the participants' in their social cognitive maps that include/exclude themselves.

Supplementary Table 3. Distribution of in-group and out-group size

	Sample 1				Sample 2				Sample 3			
**Group Size**	**Ingroup**		**Outgroup**		**Ingroup**		**Outgroup**		**Ingroup**		**Outgroup**	
**2**	41	11%	477	36%	8	7%	123	31%	48	25%	376	55%
**3**	74	20%	297	22%	16	14%	97	25%	54	28%	162	24%
**4**	53	14%	184	14%	22	19%	64	16%	47	24%	92	14%
**5**	48	13%	161	12%	19	16%	41	10%	25	13%	27	4%
**6**	46	12%	89	7%	10	9%	30	8%	10	5%	10	1%
**7**	36	10%	55	4%	17	15%	21	5%	6	3%	4	1%
**8**	35	9%	33	2%	5	4%	13	3%	3	2%	2	0%
**9**	13	4%	15	1%	4	3%	3	1%	2	1%	4	1%
**10**	6	2%	9	1%	4	3%	2	1%	0	0%	2	0%
**11**	7	2%	6	0%	6	5%	0	0%				
**12**	5	1%	0	0%	3	3%	1	0%				
**13**	2	1%	3	0%	1	1%	0	0%				
**14**	2	1%	2	0%	1	1%	0	0%				
**15**	0	0%	0	0%								
**16**	0	0%	0	0%								
**17**	1	0%	0	0%								

Note. Ingroup/out-group – groups reported by the participants' in their social cognitive maps that include/exclude themselves.

Supplementary Table 4. Cross-sample comparisons of perception accuracy

	Samples 1 vs. 2					Samples 1 vs. 3					Samples 2 vs. 3				
**Perception accuracy**	*β*	*SE*	*df*	*p*		*β*	*SE*	*df*	*p*		*β*	*SE*	*df*	*p*	
Affiliates	-.19	.03	159.80	< .001	***	-.08	.03	339.90	.014	*	.11	.04	274.28	.003	**
Non-affiliates	-.27	.03	199.01	< .001	***	-.22	.02	288.57	< .001	***	.06	.03	232.60	.074	.
Overall	-.26	.02	194.07	< .001	***	-.22	.02	275.56	< .001	***	.03	.03	224.46	.239	

Note. *β*s represent estimated mean differences; in Samples 1 vs. 2 and 1 vs. 3 comparisons, Sample 1 is used as reference category; in Samples 2 vs. 3 comparisons, Sample 2 is used as reference category.
